# Hepatic Tuberculosis Mimicking Biliary Cystadenoma: A Radiological Dilemma

**DOI:** 10.1155/2015/390184

**Published:** 2015-10-04

**Authors:** Rajaram Sharma, Amit Kumar Dey, Kartik Mittal, Prasad Udmale, Udai Singh, Sumit Mitkar, Priya Hira

**Affiliations:** Seth GS Medical College and KEM Hospital, Room No. 107, KEM Main Boy's Hostel, Acharya Donde Marg, Parel, Mumbai 400012, India

## Abstract

Primary involvement of liver in tuberculosis is a rare entity. It is difficult to diagnose in absence of previous history of tuberculosis or concurrent pulmonary involvement. It is usually misdiagnosed as neoplastic liver lesion, which misdirects the treatment protocol and delays proper treatment. Here we are presenting a case of 36-year-old male patient with vague right upper quadrant abdominal pain. All the laboratory values were within normal limits. Radiological investigations were in favor of biliary cystadenoma but final diagnosis was primary focal involvement of liver in tuberculosis which was histopathologically proven to be tuberculous granulomas on biopsy of the resected mass.

## 1. Introduction

Hepatic involvement in TB is rare; however, exact incidence is unknown, likely due to underreporting and unawareness of the disease, as most hepatic TB cases are diagnosed retrospectively upon surgery or autopsy [1]. There is recent increase in incidence of hepatic TB in the last 30 years, mostly due to increased prevalence of HIV/AIDS [2]. Mode of transmission is either hematogenous from lungs or local from intestine [3]. Hepatic involvement can be disseminated type, focal type, or cholangitic type in decreasing order of frequency of involvement [3, 4].

Here we are presenting a focal type of primary hepatic involvement. Our aim is to highlight the fact that in endemic countries like India tuberculosis should be kept in mind as a differential of hepatic mass specially in young patients. A biopsy and histopathological examination should be done before resection of the lesion.

## 2. Case Report

**Figure 1:**
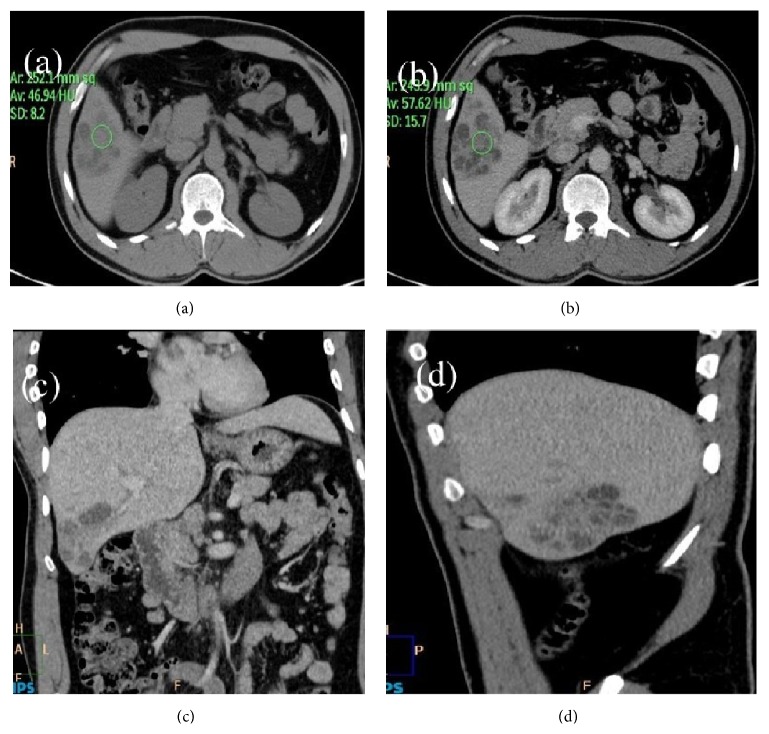
(a) Plain CT image of abdomen: axial view showing ill-defined, mixed density mass lesions in segment V of liver. Hounsfield unit at noncystic part is 47 in the center of the lesion. (b) Postcontrast CT image of abdomen: axial view showing ill-defined heterogeneously enhancing conglomerated mass lesions in segment V of liver. Hounsfield unit at noncystic part is 57 in the center of the lesion. (c) Postcontrast CT image of abdomen: coronal view showing ill-defined conglomerate mass lesions in segment V of liver. Remainder of abdomen and visualized part of lungs are devoid of any significant pathology. (d) Postcontrast CT image of abdomen: sagittal view showing ill-defined conglomerated lesions in segment V of liver. There is mild enlargement of the liver.

**Figure 2:**
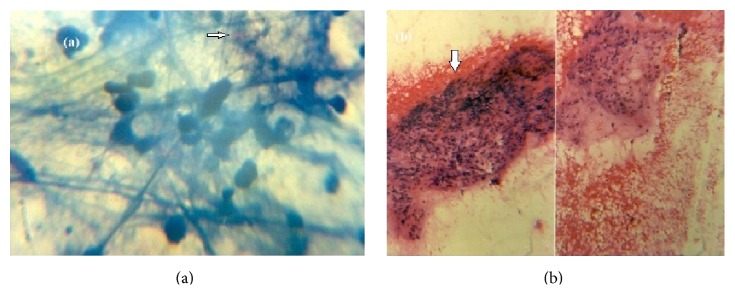
(a) Immunofluorescence using AFB (ICC, ^*∗*^100x) confirms that the antibody specifically stains one or a few mycobacteria (white arrow) inside the cytoplasm of cells. (b) On histopathological analysis (H and E, ^*∗*^100x) there was collection of ill-defined epithelioid cells, lymphoid cells, and multinucleate giant cells with central area of necrosis within (white arrow).

A 35-year-old male patient presented to our hospital with right upper quadrant dull pain since 2 months. There is no history of fever or jaundice. There are no other constitutional symptoms. USG was advised which showed enlarged liver up to 15 cm and heterogeneously hypoechoic mass lesion in segment VI of liver with peripheral vascularity. There was no organomegaly seen apart from the liver or no inflamed lymph nodes were seen. Ileocaecal junction appears normal. Chest X-ray was normal. Biochemical profile was done which showed mildly raised alkaline phosphatase. Hematological profile was normal. Tumor markers like AFP and CEA were normal. In plain CT image of abdomen, axial view showed ill-defined, mixed density mass lesions in segment V of liver (Figures 1(a), 1(b), 1(c), and 1(d)). Based on CT findings diagnosis of hepatic hydatidosis and abscess was ruled out. CT diagnosis was made of benign cystadenoma. Patient was advised surgery but he refused to be operated on. Patient presented with abdominal pain and fever again after 6 months. Imaging report on CT was similar to that of the CT scan done earlier. The patient was subjected to a surgical procedure which involved right hepatectomy. On histopathological analysis there was section of liver showing granuloma with necrosis in the centre (Figure 2(b)). Immunofluorescence using AFB confirms the histopathological findings (Figure 2(a)). Patient was discharged and is asymptomatic now after 6 months of follow-up.

## 3. Discussion

As the prevalence of tuberculosis is increasing in the world, rare presentations of the disease will be more frequently encountered. Hepatic TB is difficult to diagnose as only a few cases are reported. Diagnosis often requires biopsy, especially in focal involvement like in our case [5]. Possible reason for rarity of hepatic involvement may be low oxygen content of hepatic parenchyma [6]. There are no specific clinical signs for hepatic TB. Abdominal pain, fever, and weight loss are the common presenting complaints [7]. Hepatomegaly is the most consistent sign, which was present in our case [8]. Biochemical profile may show elevated alkaline phosphatase, while other parameters are mostly within normal limits [5]. Jaundice is a more common feature of local form of hepatic TB rather than diffuse form [9].

X-ray and ultrasound lack sensitivity and specificity in diagnosis but are still considered first line of investigations. Chest X-ray may show pulmonary tuberculosis which may be a clue for the diagnosis [10, 11]. Ultrasound may show round to oval hypoechoic lesions which is a very nonspecific sign [12, 13]. Abdominal CT is the optimal imaging modality. It shows the liver tuberculoma as a nonenhancing, low-density center lesion because of caseation necrosis with a peripherally enhancing rim relating to outer granulation tissue [14]. Multiple conglomerated lesions of varying Hounsfield unit and sizes may be seen in liver parenchyma, which is a more consistent sign. This happens because of various stages of the disease [15].

In contrast hepatic hydatidosis on CT imaging presents with a mass like hepatic lesion with irregular borders, accompanied by calcified or cystic components in the periphery of the lesion [16].

MRI is actually not of much use; however, it will show T1 hypointense lesion with hypointense rim and variable T2 appearances. On contrast imaging, it will show peripheral enhancement [17].

Resected specimen can be sent for pathological and microbiological assessment. Culture of mycobacteria is the strongest evidence of hepatic TB, but the sensitivity is very low [18]. Newer techniques like polymerase chain reaction (PCR) are more promising for diagnosing hepatic TB [18].

Hepatic tuberculosis has varied presentations and thus pathologic examination of liver lesions is essential by percutaneous fine needle biopsy which serves as an excellent diagnostic method. However, risks associated with percutaneous needle biopsy such as bleeding and tumor dissemination should be taken into consideration. PCR directly detects the presence of* M. tuberculosis* and is more useful for diagnosis of TB, but positivity rate is 57% [19]. The WHO protocol for the treatment of pulmonary TB (two months of rifampin, isoniazid, ethambutol, and pyrazinamide and then 4 months of rifampin and isoniazid) has been adopted for treatment of hepatic TB with good results [20]. The appropriate duration for treatment of hepatic TB is a matter of conflict. Usually 6–12 months duration is appropriate for most of the patients [21].

## 4. Conclusion

Primary focal involvement of liver in tuberculosis is a rare entity. It is difficult to diagnose on clinical basis and on imaging. Histopathological diagnosis is required in most of the cases. As the treatment is medical therapy, a biopsy should be done before laparotomy and excision to confirm the diagnosis to avoid a surgical misadventure, especially in endemic regions like India.
